# Supplementary material for: The Cytotoxic Properties of Some Tricyclic 1,3-Dithiolium Flavonoids
Source: Molecules. 2019 Jul 4;24(13):2459. doi: 10.3390/molecules24132459 (PMC6651846; doi:10.3390/molecules24132459)
Supplement: Supplementary file 1 [file molecules-24-02459-s001.pdf]

Supplimentary Material for

The Cytotoxic Properties of Some Tricyclic 1,3-Dithiolium Flavonoids

Laura G. Sarbu [1], Sergiu Shova [2], Dragos Peptanariu [2], Isabela A. Sandu [2], M. Lucian Birsa [1,*] and Lucian G. Bahrin[1,2,*]

[1] Alexandru Ioan Cuza University of Iasi, Department of Chemistry, 11 Carol I Blvd., 700506 Iasi, Romania; laura.sarbu@uaic.ro (L.G.S.); lbirsa@uaic.ro (M.L.B.)

[2] Petru Poni Institute of Macromolecular Chemistry, Intelcenter. 41A Grigore Ghica Vodă Alley, 700487 Iasi, Romania; shova@icmpp.ro (S.S.);sandu.isabela@icmpp.ro (I.A.S.); peptanariu.dragos@icmpp.ro (D.P.); bahrin.lucian@icmpp (L.G.B.)

* Correspondence: lbirsa@uaic.ro (M.L.B.); bahrin.lucian@icmpp (L.G.B.)

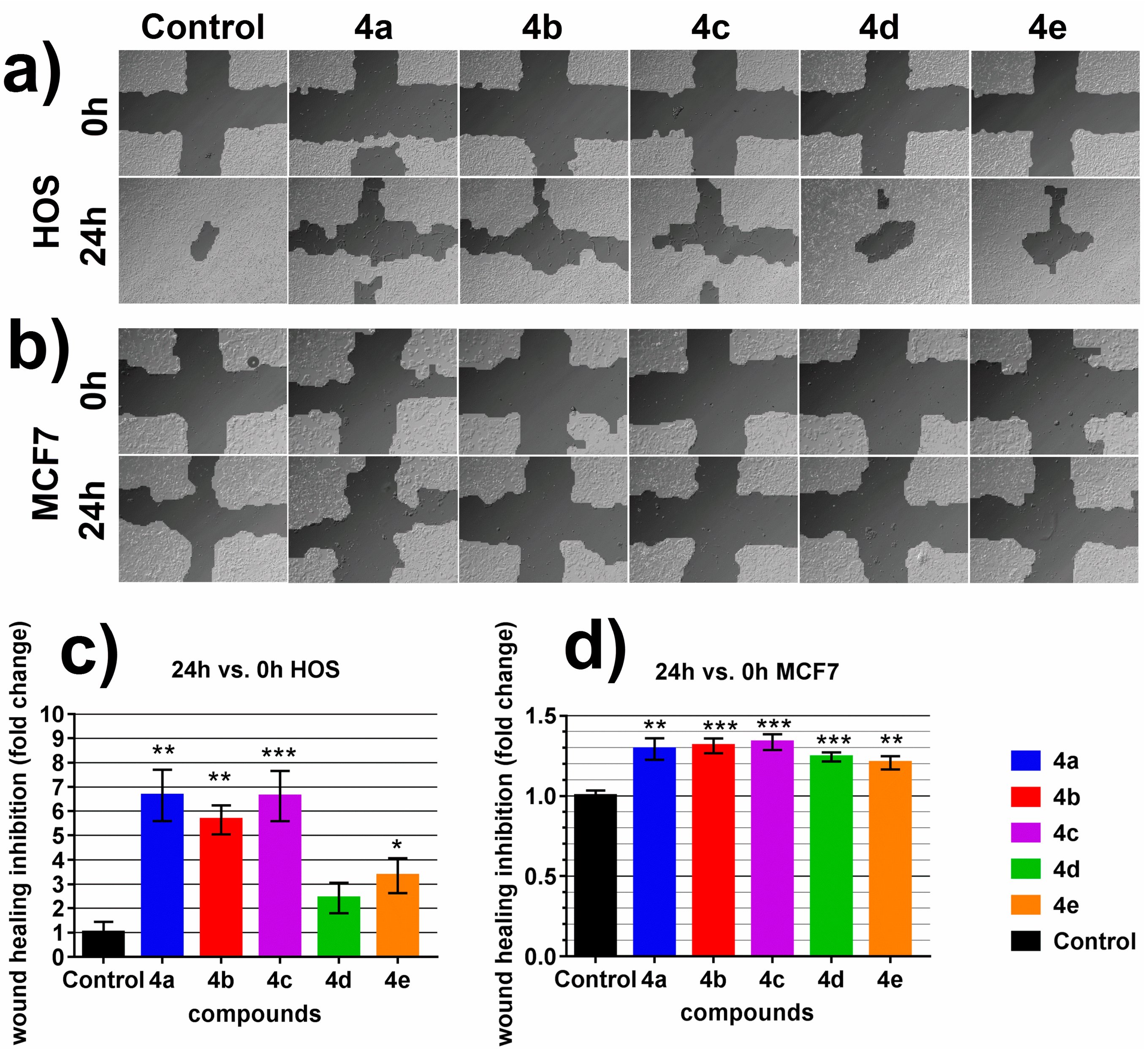

Figure S1 - Wound scratch assay. Graphs represent ratios between treated samples and control percentages of the initial open area which remained uncovered by cells after 24 hours. The results are presented as a mean value ± the standard error of the mean (S.E.M.), n = 3–8. * $p < 0.05$, ** $p < 0.01$, and *** $p < 0.001$ treated vs. control.
